# Preservation potential of keratin in deep time

**DOI:** 10.1371/journal.pone.0206569

**Published:** 2018-11-28

**Authors:** Mary Higby Schweitzer, Wenxia Zheng, Alison E. Moyer, Peter Sjövall, Johan Lindgren

**Affiliations:** 1 Department of Biological Sciences, North Carolina State University, Raleigh, North Carolina, United States of America; 2 North Carolina Museum of Natural Sciences, Raleigh, North Carolina, United States of America; 3 Department of Geology, Lund University, Lund, Sweden; 4 Department of Biology, Drexel University, Philadelphia, Pennsylvania, United States of America; 5 RISE Research Institutes of Sweden, Chemistry and Materials, Borås, Sweden; Institute of Materials Science, GERMANY

## Abstract

Multiple fossil discoveries and taphonomic experiments have established the durability of keratin. The utility and specificity of antibodies to identify keratin peptides has also been established, both in extant feathers under varying treatment conditions, and in feathers from extinct organisms. Here, we show localization of feather-keratin antibodies to control and heat-treated feathers, testifying to the repeatability of initial data supporting the preservation potential of keratin. We then show new data at higher resolution that demonstrates the specific response of these antibodies to the feather matrix, we support the presence of protein in heat-treated feathers using ToF-SIMS, and we apply these methods to a fossil feather preserved in the unusual environment of sinter hot springs. We stress the importance of employing realistic conditions such as sediment burial when designing experiments intended as proxies for taphonomic processes occurring in the fossil record. Our data support the hypothesis that keratin, particularly the β-keratin that comprises feathers, has potential to preserve in fossil remains.

## Introduction

The vast majority of data from extinct vertebrates derive from biomineralized remains (e.g. bones and teeth). Nonetheless, in exceptional fossils, originally unbiomineralized “soft tissues” have been reported, even from invertebrate fossils, dating to at least the Silurian (e.g. [1]); this suggests that natural mechanisms exist to stabilize these materials before degradation is complete [2]. Whether this exceptional morphological preservation extends to the molecular level has not, in most cases, been rigorously tested. In part, this is because existing models of fossilization assume extensive diagenetic alteration, such that original components are completely degraded or unrecognizable in their fossil form [3]. This supposition is based upon kinetic models or extrapolation of small data sets to the larger fossil record (e.g. [4–7]). However, models are hypotheses, and thus subject to testing. Empirical data are more valid than models, and can be used to either support or overturn them. We have shown, by both erecting and testing hypotheses through actualistic experiments (e.g. [8–11]) and by deriving data from fossils (e.g. [12–25]), that common assumptions about fossil preservation may be incomplete or inaccurate. Our current understanding of tissue and molecular preservation does not take into account factors that may influence preservation, such as association with mineral (e.g. [26]) or post-depositional binding of molecules to exogenous or endogenous organics [27] that can act to stabilize molecules across geological time.

The most common occurrence of “soft tissue” preservation is that of the integument, and keratinous structures (e.g. scales or feathers) derived from it ([28] and references therein). Fossilized integumentary structures derived from keratinous materials have been noted in the literature since at least the middle of the 19^th^ century (e. g. [29]). Integumentary remains preserved with exquisite articulated specimens have been used to erect and/or test phylogenies [30, 31] based upon characters not discernable from osteological evidence alone.

Keratinous structures preserved in fossils have also been used to infer the origin of evolutionary novelties. For example, the discovery of an articulated *Archaeopteryx* specimen with feather impressions in a calcareous matrix [32, 33] provided a major advance for the then-new theory of evolution by natural selection [34]. Today, non-avian and avian dinosaur fossils preserved with feathers are known from many specimens within different taxa (e.g. [35–40] and references therein).

Despite recent claims that keratin has low preservation potential [41, 42], here we add to the evidence already existing that this protein is exceedingly robust, particularly the β-keratins comprising mature, extant feathers [43–46]. We build on previous work examining the molecular and microstructural characteristics of modern feathers exposed to degradation under varying conditions for seventeen years (ten years of experimental conditions, including constant high heat, then seven additional years, where samples were stored in burial sediments or in sterile, 1.5 ml tubes, at room temperature until collected for the current analysis [10]). We then apply these methods to relatively recent ‘fossil’ feathers, recovered from a previously described, approximately 10 Ka coot (*Fulica americana*), preserved in siliceous hot spring deposits [47].

## Materials and methods

Feathers taken from a single specimen of Hungarian (gray) partridge (*Perdix perdix*) were subjected to three different burial conditions for a period of 10 years, then recovered and kept for an additional seven years at room temperature until analyzed (see [10] and S1 File for detailed discussion). Briefly, feathers were (1) buried in sands taken from the Judith River formation (Montana, USA), watered intermittently with distilled water, and incubated at 60°C for three years, then allowed to dry, but kept buried at room temperature (RT) until analyzed (*condition 1*); (2) buried, covered and (dry) heated to 350°C for 10 years (in a sterilization oven used for microbiological processing, and in continual use), then stored, still buried, or in sealed, sterile 1.5 ml tubes at RT an additional seven years until analyzed (*condition 2*); (3) kept covered but unburied at RT for the full 17 years (*control*).

To test the durability of keratin epitopes (small regions of a protein to which an antibody binds), we included feathers from an unusually preserved, three-dimensional specimen of American coot (*Fulica Americana*, National Park Service Accession number YELL 147421, ~10Ka), entombed in hot springs sinter deposits from Yellowstone Park, USA [47]. This specimen was preserved in a naturally occurring, but extreme environment, and the high heat and low pH experienced by these feathers adds support to the durability of keratin we show in our experiments. The feathers were encrusted in silica (identified by X-ray diffraction as opal-A), and initially identified as external molds [47].

Re-examination of the experimental feathers using transmission (TEM) and scanning (SEM) electron microscopy, time of flight secondary ion mass spectrometry (ToF-SIMS), and *in situ* immunology [immunofluorescence (IF) and immunogold (IG)], show ultrastructural preservation in these feathers, and support the hypothesis that at least some original organic material consistent with protein remains associated with these tissues even after exposure to extreme conditions.

Time-of-flight secondary ion mass spectrometry (ToF-SIMS) was used for molecular characterization of the 350°C (*condition 2*) and *control* feathers. ToF-SIMS is a mass spectrometry method capable of identifying and localizing molecular species to solid surfaces [48, 49]. Thus, this method can identify amino acids and small peptides (up to ~1500 Da), and localize them to specific tissues with high resolution; however, it does not specifically identify source proteins or sequence.

A focused, high energy (primary) ion beam was directed onto the sample surface, causing secondary ions to be emitted, resulting in mass spectra containing molecular information. Spatial information was obtained by scanning the primary ion beam over a specified analysis area and recording separate mass spectra in each pixel, allowing the generation of ion images that display the signal intensity of specific molecular signals across the analysis area or of mass spectra of specific regions of interest. For further details of methods and sample preparation, see S1 File.

## Results

### Light microscopy

**Fig 1:**
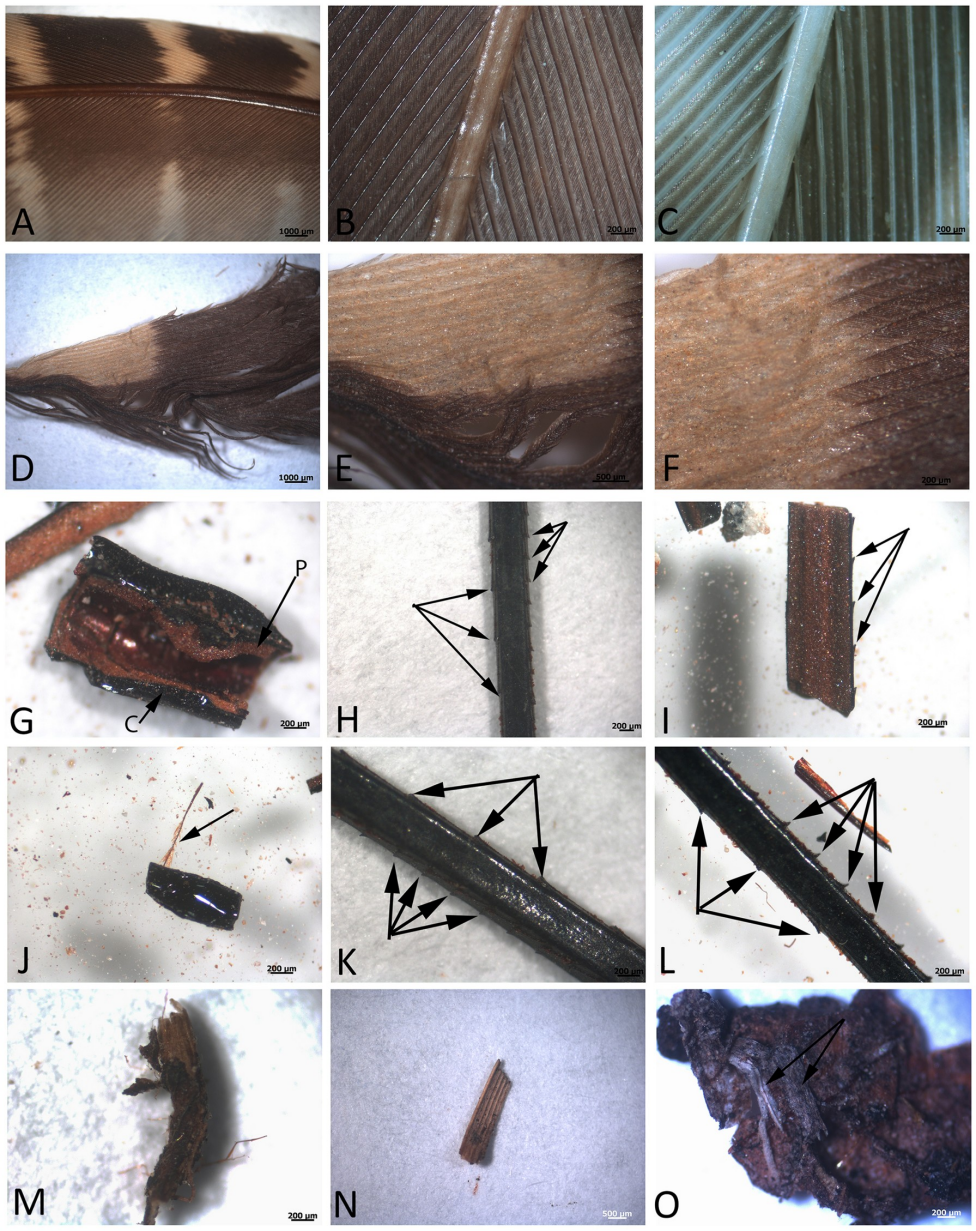
Light micrographs (LM) of feathers used in this study. A-C are control feathers, kept at room temperature (RT) for the 10-year duration of the experiment, and additional six years until present analyses. A) overview, showing original color distributions of feathers from *Perdix perdix*. B) red, and C) white regions of the feather. D-F show the *condition 1* feather, kept at 60°C with intermittent watering for 3 years, then buried at RT until analyses. Colored regions are still distinct, but barbs show fraying and loss of integrity. Panels G-L show fragmented remains of feathers buried and maintained at 350°C for ten years, then at RT until analyses. No evidence of original color remains; barbules are not in evidence (but see J, arrow). G) shows a region of hollow rachis, with pith (p) internal, and a dark carbonized external cortex (c). H) Shows a region of the rachis (identified by diameter comparisons with A-C) preserved in three dimensions. Although no original color remains, the offset barb ridges (arrows) allow us to determine this shaft is from a remige (flight feather). I) is a longitudinal section through the shaft. Barb ridges can be seen on one side of this structure (arrows) and a lighter colored pith is visible filling the rachis. J) shows a tiny barbule with presumed hooklets arising from it (arrow) associated with a small fragment of a feather rachis. K) and L) represent other rachises (or parts) preserving the offset barbs intact and in three dimensions (arrows). M-O represent the remnants of a silicified coot feathers, collected from Yellowstone National Park. We interpret M) to be a degraded feather rachis, displaying fibrous surface. N) A small region interpreted to represent overlapping barbs, forming a vane. Additional fibrous remnants still embedded in silicified coated region can be seen in panel O (arrows).

Feathers exposed to the above conditions were examined using light microscopy (LM; Fig 1). No apparent alteration can be detected from the living state in the *control* feather (Fig 1A–1C); both pigmented (Fig 1B) and non-pigmented (Fig 1C) regions appear unaltered. Fig 1D–1F shows LM images of feathers from the same bird under test *condition 1* (60°C, watered). Twisted barbs, fraying, and slight loss of integrity can be observed, but the feathers remain virtually intact, with little evidence of damage at this relatively low resolution. However, the colors are less distinct; “white” regions (Fig 1F) show evidence of yellowing, perhaps from oxidation or pigment leaching, when compared to the control feathers at the same magnification (Fig 1C). The feathers kept under *condition 2* (high heat, Fig 1G–1L) have completely lost all indications of original color or color patterns, and are reduced to blackened fragments. Surprisingly, however, the microstructure remains. The rachis is hollow and preserved in three dimensions. Remnants of pith (p) can still be detected as lighter colored, textured regions, distinct from the darkened cortex (c) (Fig 1G). Barbs can be seen arising from a preserved rachis (Fig 1H, 1I, 1K and 1L —arrows). The asymmetric distribution (Fig 1H, 1K and 1L) allows us to state that these are remnants of remiges (flight feathers). In some cases, isolated barbules with presumed hooklets are retained as long, filamentous structures (Fig 1J—arrow). Light micrographs of silicified fossil coot feathers are seen in Fig 1M–1O. The fibrous structure is evident in feathers still attached to the siliceous matrix (Fig 1M–1O), and Fig 1N shows a small region where overlapping barbs form a vane that is seemingly intact.

### Scanning electron microscopy

**Fig 2:**
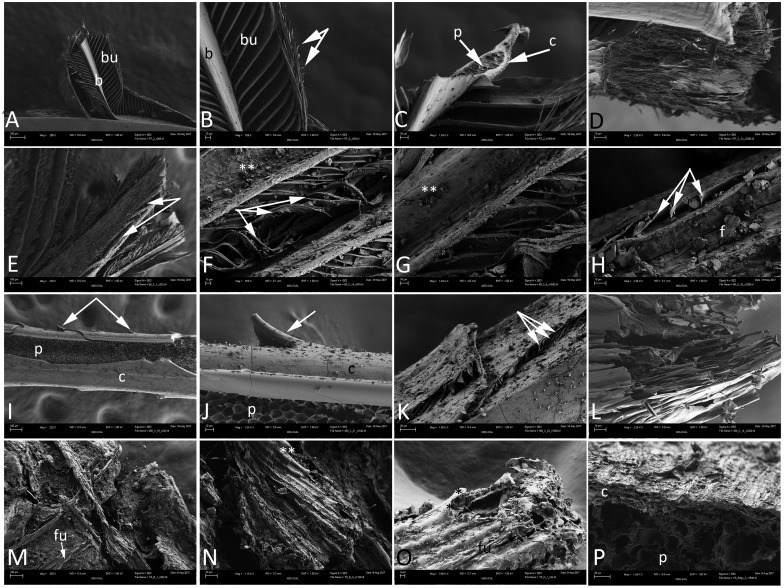
Scanning electron micrographs (SEM) of experimental (A-L), and Yellowstone (M-P) feathers. A-D) barbs arising from a rachis at low (A) and higher (B-D) magnifications. Feather structure is virtually unaltered, and both barbs (b) and barbules (bu) can be seen. In Fig 2B, hooklets are seen arising from distal bars (arrows). C) Internal regions of a barb, with cortex (c) and pith (p) clearly discernible. D) Highly fibrous region of what is interpreted to be the distal rachis. E-H represent the *condition 1* feathers. Loss of integrity is more obvious than in LM. E) Fraying of the rachis (arrows) reveals fibrous structure. F) Twisted and compressed barbules (arrows) and debris on the rachis and barbs (**). G) Higher magnification of rachis and barbs, with debris (**) that may be from the burial sands, or from degrading keratin flakes. H) Bent and twisted barbules (arrows) with presumed keratinous flakes (f) on the surface of feather structures. Panels I-L show the microstructural integrity of the *condition 2* (350°C) feather. I) Rachis, with smooth external cortex (c) and internal pith (p). Barbs are seen arising from the surface (arrows). J) Higher magnification image showing pith (p) and cortex (c), but the cortex demonstrates thin cracks in the surface. A curved barb is still attached (arrow). K) Compressed barbules (arrows) arising from flattened barbs.; debris can be seen across the surface of these feather structures. L) Highly fibrous region of the *condition 2* feather, very similar in structure to that seen in the control (D). Panels M-P show the three dimensional, coated structure of the silicified coot feather. M) Fibrous structure and overlapping barbs in low magnification, with evidence of fungal hyphae (fu) interspersed throughout. N) Region of overlapping barbs, with thin mineral coating (**). O) Thin mineral coating on the barbs in higher magnification (**); silicified fungal hyphae can also be seen (fu). P) Feather at higher magnification, revealing a fibrous outer cortex (c), and altered pith (p) interior to the cortex. Scale bars: A, E, I, M are 100 μm; K, N, P = 20 μm; O = 3 μm; all others = 10 μm.

Scanning electron microscopy (Fig 2) demonstrates that all feathers are preserved in three dimensions. Feather microstructure is essentially intact under *control* conditions (Fig 2A–2D). Barbs (b) and barbules (bu), some with hooklets (Fig 2B—arrows) can be seen. Inner pith (p) is easily differentiated from the feather cortex (Fig 2C—arrows), and structure of the original keratin fibers can be seen in some regions of the feather internal to the proximal cortex after 17 years (Fig 2D). Under *condition 1*, fraying and disintegration of the barbs and barbules, as well cracking and loss of integrity of the cortex is visible (Fig 2E—arrows). Barbs are distorted (Fig 2F), and debris is visible on barbs and barbules (Fig 2F and 2G —**), but in some cases hooklets can still be seen at the ends of the barbules (Fig 2F—arrowheads). Fig 2H shows curved barbules coming off the barb (arrows), and a surface littered with flattened plates/flakes of material (f).

Preserved and intact microstructure in *condition 2* feathers from the 10 year-experiment is seen in Fig 2I–2L. The cortex (c) and pith (p) are clearly visible and distinct (Fig 2I and 2J), and small remnant barbs can be seen, evenly spaced and arising from the cortex (arrows). Fragmented and compressed barbules can be seen arising from barbs (Fig 2K—arrows) and fibrous structures (Fig 2L) very similar to those seen in the *control* feather (Fig 1D) are visible.

Fig 2M–2P show silicified feathers recovered from the fossil coot. Fungal hyphae (*fu*) and other debris are intertwined with the feather barbs (Fig 2M). Although a thin layer of precipitated minerals is seen (Fig 2N and 2O—**), underlying feather structure is still visible in detail. What we interpret to be remnant feather cortex (c) and underlying pith (p), are visible in Fig 2P.

### Immunohistochemistry

**Fig 3:**
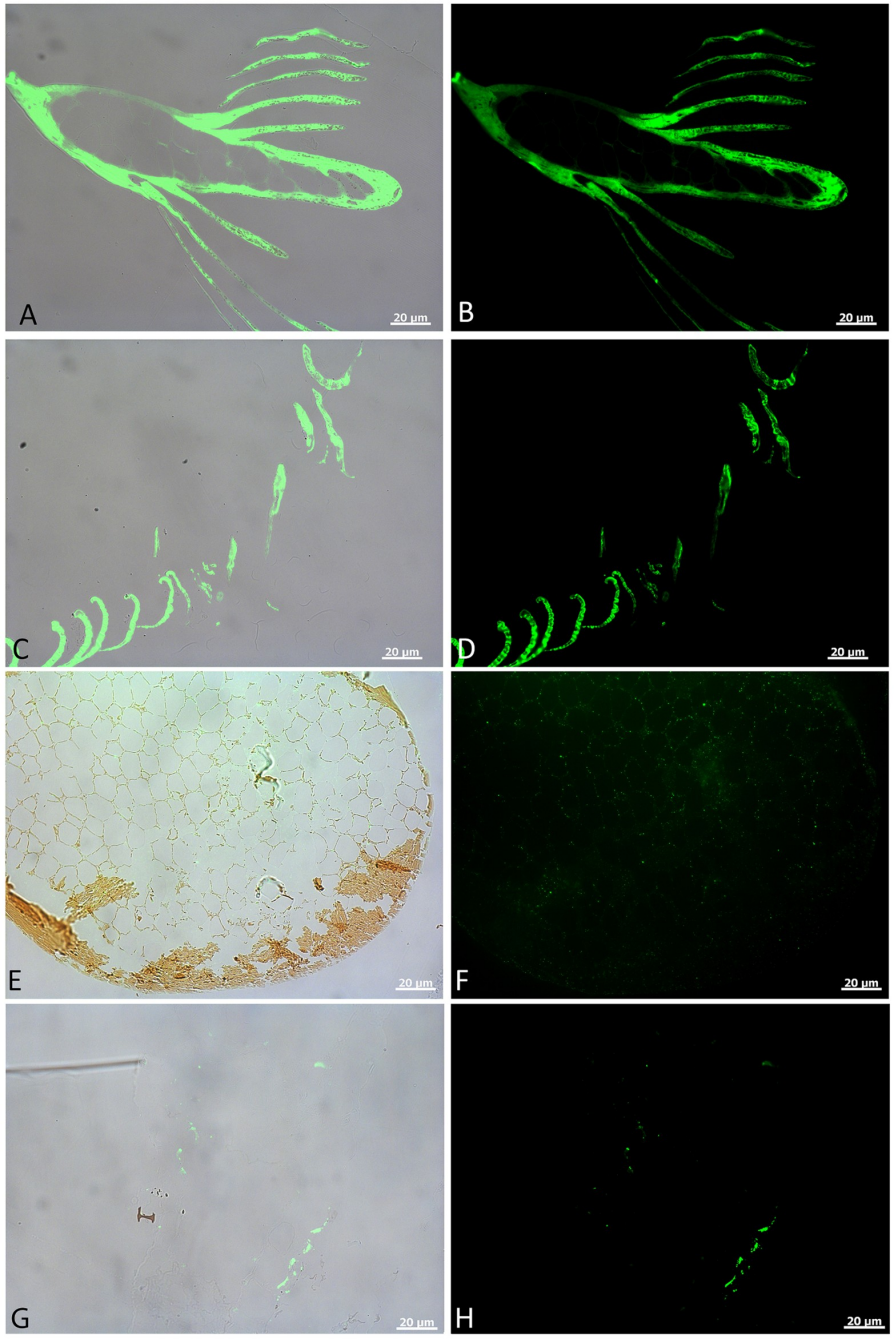
*In situ* immunofluorescence on feather tissues. A, C, E, and G are overlay images; B, D, F, and H are fluorescence images, showing localized binding of antiserum raised against modern feathers to these experimental feathers. A, B) show *in situ* binding of the serum to feather rachis and barbs in *control* feathers. Antibody-antigen (ab-ag) complexes are demonstrated by localized green signal under fluorescent light. C, D) Virtually undiminished binding of antibodies to the *condition 1* feather barbs. No spurious binding is seen on the embedding polymer, and ab-ag complexes are specific to feather structures. E, F) Cross section of a feather barb from *condition 2*. A thin cortex can be seen, with very thin rami of pith in E). F) Weak, but highly localized binding of antiserum to feather structures, with no binding observed outside of the tissues. G, H) Localization of ab-ag complexes to the surface of tissues seen in the Yellowstone feather. Binding is restricted to feather structure, as can be seen in G, but is intermittent and, although structurally preserved, not all feather material binds this antiserum.

Affinity-purified antibodies (abs) raised against chicken feather protein [10] react specifically with all feathers tested in *in situ* immunohistochemical assays (Fig 3). Overlay (Fig 3A, 3C, 3E and 3G) and fluorescent images (Fig 3B, 3D, 3F and 3H) show that antibody-antigen (ab-ag) complexes localize specifically to the feather tissues, and are not randomly distributed. Specificity of binding is supported by controls, including: (1) omitting primary abs, but keeping all subsequent steps identical to control for non-specific binding of the secondary ab or fluorescent label (S1 Fig and [10]); (2) Incubating antibodies with excess source protein (e.g., feather keratin) to block specific binding sites, then exposing them to samples controls for non-specific binding of primary antibody [10]; (3) incubating feather specimens with a non-relevant protein (human alpha keratin, [10]) controls for non-specific binding of primary antibodies. Although binding is greatly reduced and patchy in the *condition 2* feather (Fig 3E and 3F) relative to *control* and *condition 1* feathers, it is specific, not observed where tissue is lacking; this pattern compares favorably with previously published results [10], showing repeatability and thus validating original results. Fluorescent signal is also reduced in fossil feathers (Fig 3G and 3H) when compared to the less altered *control* (Fig 3A and 3B) and *condition 1* feathers (Fig 3C and 3D), but binding is nonetheless both specific and highly localized to microstructural features of the feathers.

### Transmission electron microscopy (TEM)/Immunogold (IG) labelling

**Fig 4:**
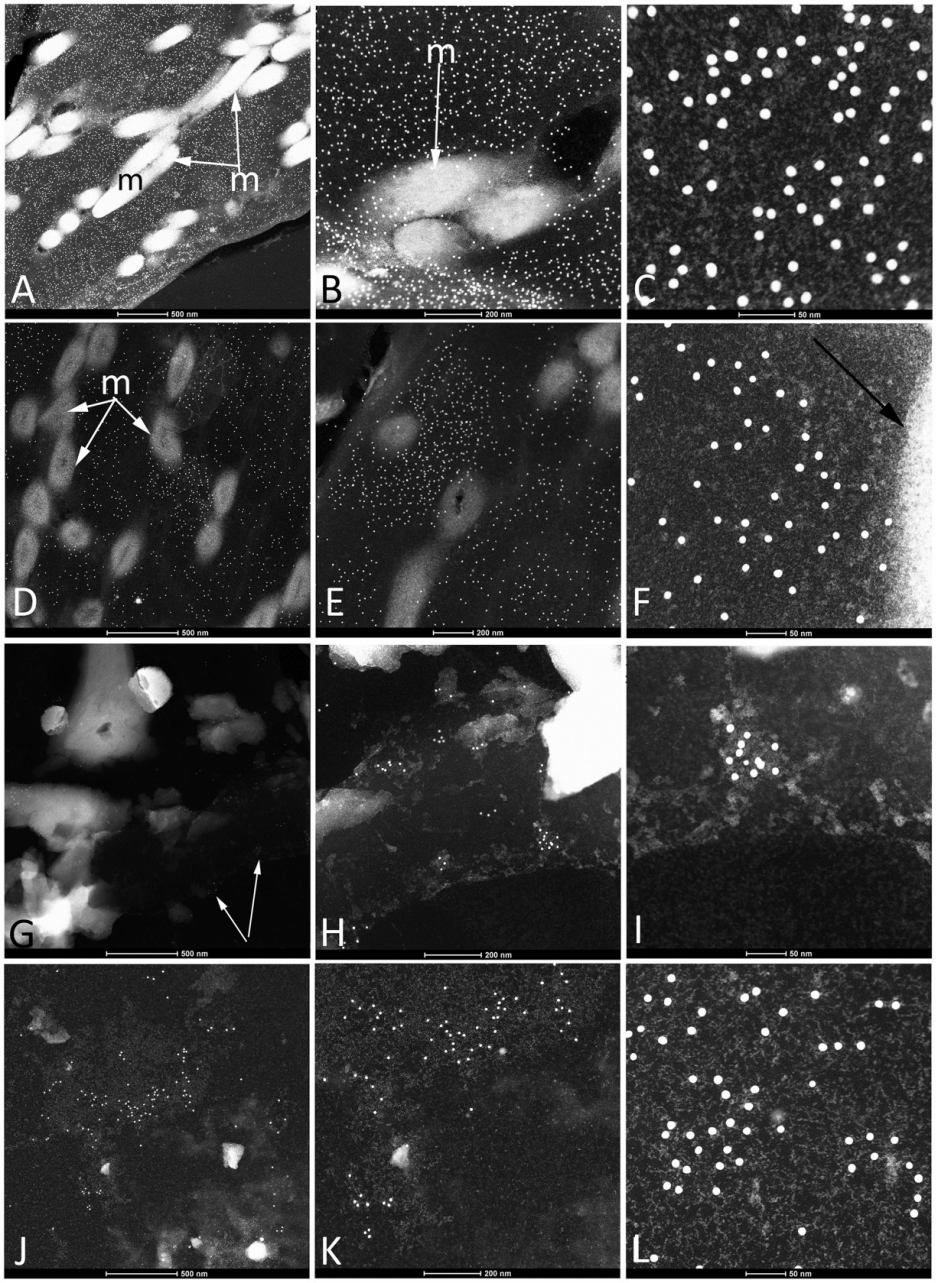
Transmission electron micrographs (TEM) and immunogold labeling of experimental and fossil tissues with antiserum to feather keratin. Ab-ag complexes are demonstrated by electron-opaque gold beads attached to the secondary antibody. A-C) Localization of ab-ag complexes to keratinous tissues in the *control* feather. Melanosomes can be seen in A, B), but virtually no gold beads localize to these structures, and remain localized only to the filamentous matrix, supporting antibody specificity. C) gold beads are specifically associated with electron-lucent filaments against a darker background. D-F show the same immunoassay results on the *condition 1* feathers. Melanosomes can be seen, but they are less electron dense than in A), and most exhibit hollow cores, possibly indicating initial degradation. Again, gold beads, reflecting the location of ab-ag complexes, are localized to the keratinous matrix interspersed between melanosomes, although these are reduced in density from the *control* feathers. F) Edge of a melanosome (arrow); no binding of the small gold beads is observed on the melanosome, but is localized to the matrix surrounding the melanosome. G-I) Immunolabelling on a small region of the *condition 2* feather; although no melanosomes are seen in feathers from this condition, the keratinous matrix remains. Binding of antibodies is sparse, but is specific and highly localized to remnants of electron-lucent filaments (H, I). J-L) Localization of gold beads to Yellowstone coot feathers under low (J) and higher (K, L) magnification. No melanosomes are visible in TEM, but gold beads are strictly localized to regions of small electron lucent filaments, as in the other conditions presented here.

Extant feathers under all three conditions and the mineralized coot feather are shown in Fig 4 after exposure to antibodies raised against feather keratin. Here, we use a secondary antibody tethered to a small (18nm) electron-dense gold bead, to detect antibody-antigen complexes *in situ* at high resolution. At all levels of magnification, gold beads are clearly visible on the keratin matrix of the *control* feather kept at RT (Fig 4A–4C), and very few are found on the surface of electron-dense melanosomes visible in the same sections. These data support the specificity of these antibodies, and show that they can be used to distinguish between tissue types. Intact melanosomes are seen embedded in and surrounded by a keratinous matrix, to which the gold-labeled antibodies bind exclusively (Fig 4C).

The *condition 1* (60°C) feather (Fig 4D–4F) shows a similar pattern; however, the melanosomes have lost electron density when compared to the control, and exhibit hollow regions at their centers, not seen in the control condition. These features may indicate beginning breakdown and loss of melanin from these organelles, or alternatively, increasing electron density of the keratin matrix resulting in less contrast between organelle and matrix. Gold beads are seen between melanosome bodies and localized to the matrix, confirming specific binding as demonstrated in Fig 3. At higher magnifications (Fig 4F), the matrix is again seen to be filamentous, as indicated by the short and interwoven electron-dense fibers. The edge of a melanosome is seen in Fig 4F (arrow) but no gold beads are associated with this structure.

Fig 4G–4I are increasing magnifications of the *condition 2* feather, kept at 350°C. No melanosomes are observed, but antibody-antigen complexes are visible, though reduced in density from *condition 1* and *control* feathers (Fig 4G—arrows). Gold beads are seen interspersed on the filamentous structures, and are not randomly distributed. Higher magnification (Fig 4I) shows the same filamentous pattern to the matrix as seen in previous examples; the filaments are less distinct than in the *control* and *condition 1* samples but ab-ag complexes are specifically associated with microstructures and again, not random in distribution. However, not all visible filaments bind antibodies, testifying to loss of antigenicity in most of the keratin fibers.

The silicified feather from the Yellowstone coot shows a similar filamentous, electron lucent, but patchy background (Fig 4J–4L). The antibodies bind in a pattern similar to the 350°C feather, in that binding is non-random, and less concentrated than in the *control* and *condition 1* feathers (Fig 4A–4F), but it is specific and distributed only to the filamentous matrix. No obvious melanosomes are seen in this feather; this may be due to complete degradation, or it may be that the original feather never had them, and was white in color. However, extant coots are darkly pigmented, so the latter is unlikely.

The *condition 2* and coot feathers were also visualized under lower magnification TEM to identify ultrastructural similarities remaining after induced (*condition 2*) and natural (coot) heat degradation. S2 Fig shows that both retain open, thin-walled pith, although there is some distortion in the coot. Both show filamentous structures that are thicker at the pith wall junctions, but laminae are seen in the coot (S2C and S2D Fig) that are not so apparent in the *condition 2* feather (S2A and S2B Fig).

### Time of flight secondary ion mass spectrometry (ToF-SIMS)

ToF-SIMS was used for molecular characterization of the 350°C (*condition 2*) and (*control*) feathers, to compare preservation (or loss) of proteinaceous structures in the 350°C feather relative to the *control*. Although *specific* proteins (e.g. keratin) cannot be identified in ToF-SIMS, material consistent with protein/peptides are recognized by the presence of fragment ion peaks in mass spectra that represent various amino acids [50]. In particular, positive ToF-SIMS spectra of proteins are often dominated by peaks corresponding to nitrogen-containing organic fragment ions, such as CH_4_N^+^ (m/z 30), C_2_H_6_N^+^ (m/z 44), and C_4_H_8_N^+^ (m/z 70) [51].

**Fig 5:**
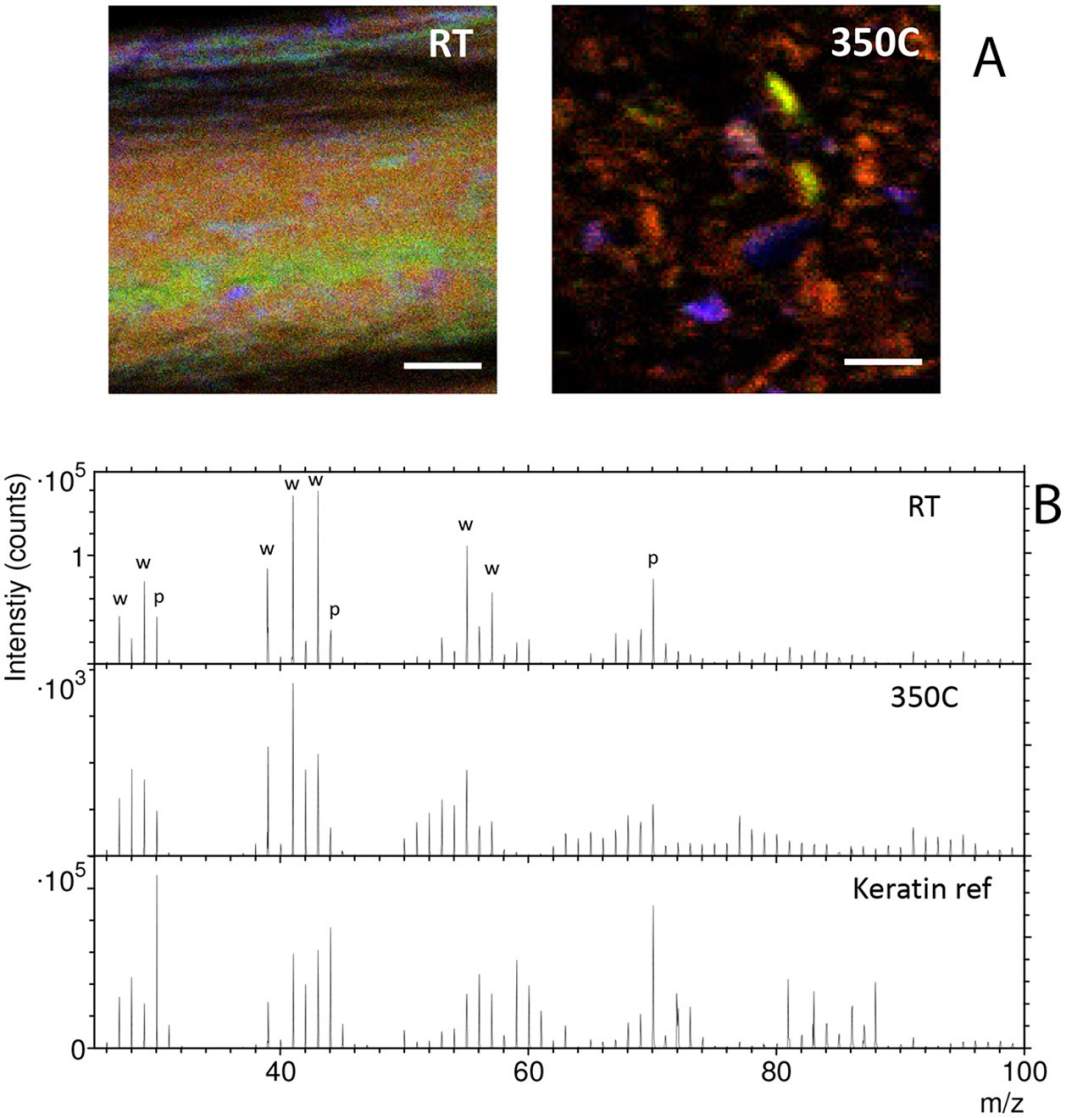
Positive ion ToF-SIMS data of the *control* (RT) and *condition 2* (350 °C) feathers, keratin reference sample and tape support. A) Ion images show the signal intensity distributions of C_4_H_8_N^+^ (green), representing protein, C_5_H_9_^+^ (red), representing hydrocarbons, and Ca^+^ +CaOH^+^ (blue). Scale bar 100 μm. B) Mass spectra generated from the protein-rich areas of the control and 350°C feathers, a keratin reference, and the tape substrate onto which the feather samples were attached (see text). Strong peaks at m/z 70, 30 and 44 in the keratin spectrum and the relatively strong signal of these peaks also in the feather spectra are consistent with proteinaceous material in both feather samples.”w” indicates hydrocarbon ions, and”p” indicates nitrogen-containing ions that show strong intensity for proteins. Optical micrographs of the feather samples are provided in S6 Fig.

ToF-SIMS was used to investigate the presence of proteins in the *control* and 350° (condition 2) feathers. The ToF-SIMS data from both feathers share features in common with the spectrum of a keratin reference sample (K0253, Sigma-Aldrich), including strong signal from the protein peaks at m/z 30, 44 and 70, consistent with the presence of proteinaceous material on both feather surfaces (Fig 5). However, neither feather sample produced ToF-SIMS spectra that in detail mimic the keratin spectrum, indicating that other molecular components may also be contributing to the spectra. This difference can be explained for the *control* feather in part by the presence on the feather surface of not only keratin but also of preen waxes, which are used by birds to protect their feathers from environmental influences [52–54]. These waxes are identified in the negative ion spectra as monoester molecular ions in the mass range m/z 290–460 and diester molecular ions at m/z 680–800 (S3 Fig), corresponding to the main components of bird feather preen waxes [52]. The assignment to preen waxes is based on the exact mass agreement of the observed peaks with reported molecular weights of the most abundant monoesters and diesters in red knot [52, 53] and herring gull [53]. The presence of preen waxes on the control feather is also reflected in the positive ion spectrum by enhanced signal from hydrocarbon fragment ions (m/z 27, 29, 39, 41, 43, 55, 57), which are not present at the same high intensities in the pure keratin reference spectrum (Fig 5). The presence of keratin in the *control* feather is further supported by enhanced protein signal along the longitudinal (green) area of the three-color overlay image (Fig 5A), which is matched by reduced signal from the hydrocarbon fragment ions (red) and from the monoesters (S3 Fig), suggesting that this area was generated by the removal of preen wax while pressing the feather sample during sample preparation. The peaks assigned to preen waxes were only observed for the control feather, and not for the heat-treated feather under *condition 2*.

The *condition 2* feather, like the control feather, was crushed to access interior surfaces not likely to be subject to environmental contamination. Enhanced signal from protein peaks at m/z 30 and 70 is observed primarily in certain regions, whereas other regions are devoid of these protein signals (Fig 5, S4 Fig), indicating that proteins (i.e., keratin) were better preserved in some structures, but effectively decomposed in others, consistent with all immunological data. The negative ion data of the 350°C feather is dominated by N-containing organic fragment ions indicative of a nitrogen-rich organic material, as expected from proteins degraded without loss of nitrogen (S5A Fig). The presence of degraded proteins is further supported by the observation of a broad distribution of peaks in the mass range m/z 100–500 (S5 Fig), corresponding to protein fragments of various sizes, and by the enhanced signal from organic fragment ions with low hydrogen content, indicating loss of hydrogen, as compared to the keratin reference spectrum (Fig 5). The ToF-SIMS results of the 350°C feather are thus consistent with keratin being effectively degraded in the main part of the feather, but also relatively well preserved in certain structures.

## Discussion

Actualistic taphonomy is important for accurately interpreting data from the fossil record. Understanding the processes of degradation in natural environments allows us to constrain, within reason, conditions that may arrest these processes or, alternatively, that may result in stabilization of fossil remains before degradation is complete [2]. If stabilization does not occur, degradation goes to completion, and all evidence of organic remains is lost, as happens in the vast majority of cases.

“Soft” (i.e., originally unbiomineralized) tissues in ancient fossil material provide information about past life that is disproportionate to their occurrence. The longevity and high preservation potential of keratin is supported by the fact that keratin-derived structures are second only to biomineralized remains (bone, teeth and eggshell) in the vertebrate fossil record ([28] and references therein), indicating that this protein is capable of resisting degradation long enough for these materials to enter the rock record. The processes that result in this preservation are not completely known, and probably vary with source tissues and burial environments; however, certainly part of this resistance is imparted by the structure of the β-keratin molecule itself.

β-keratins are specific to sauropsids, arising after the divergence of mammals from this lineage [44, 46, 55–59]; therefore, mammals employ only α-keratins in epidermal structures. Multiple studies have capitalized on the specificity and sensitivity of the vertebrate immune system, using antibodies capable of differentiating between keratin proteins to study regional expression of β-keratin [43, 46, 55, 60, 61], expression during development [45, 62–64] and co-expression of α- and β-keratins in various tissue types [43, 60, 61].

Far from being “impossible” [41], we have validated and added to a previous study [10] supporting the durability of β-keratin proteins and their potential for preserving across geological time. We replicated previous experiments, showing that macro- and microstructure is conserved to some degree in these heat-altered materials, using transmitted light, scanning and transmission electron microscopy. We employed *in situ* immunohistochemistry (IHC) to support the hypothesis that high heat, often used as a proxy for time in degradation studies (e.g. [4, 65]), is insufficient to completely destroy the molecular and microstructural characteristics of these tissues, even over long durations, although the molecules are greatly altered, as evidenced by reduced, though still specific, binding of antibodies to tissues. We support these IHC data using immunogold labelling to localize antibody-antigen complexes at very high resolution, and employ ToF-SIMS to support the presence of protein moieties localized to these tissues.

We have also demonstrated that, at least for samples subjected to the conditions described herein, keratin seems to have higher preservation potential than melanosomes, which are not seen in TEM of either the feathers exposed to high heat (*condition 2*) or the coot feathers. We did not test for the presence of melanin pigment, and it may be present; but melanosomes, the intracellular organelles used to propose color in extinct organisms (e.g. [66–68]) were not observed. It may be that both specimens preserved only feathers lacking melanosomes to begin with, but extant coots are darkly pigmented, and most of the feathers of the partridge were originally colored; this seems an unlikely explanation for the absence of these organelles. Therefore, when microbodies or imprints of microbodies proposed to be melanosomes have been identified in fossil feathers, the chemical identification of keratin may independently support the identification of these pigment-containing organelles in fossil remains even when no melanin is chemically identified.

However, keratin preservation under extreme conditions is uneven. A possible explanation for this apparent selective preservation may be related to macromolecular aggregation and hydrophobic interactions, as previously proposed for protein preservation in marine systems [69]. Macromolecular crosslinking, mitigated by certain microenvironmental factors (e.g iron, [70]), may also play a role in this selective preservation, as may the structure of feather keratin molecules, which incorporate multiple crosslinks and hydrophobic residues ([71], [72] and references therein) that contribute resistance to degradation. Finally, although most keratins are not normally biomineralized in life (contra [41] and cited references), and extant feathers have not been shown to contain biominerals, keratins are negatively charged [73, 74]; thus post-mortem micromobilization of minerals may deposit on the surface, and may contribute to the stabilization of these molecules [16, 17].

## Conclusions

We have shown, repeatedly and using multiple lines of evidence [16, 17, 75, 76], that sauroposid β-keratin products are extremely resistant to degradation under some conditions, and survive at the molecular level such that they are recognizable by specific antibodies over geological time. This resistance is not limited to tissue type [77]; thus, antigenic properties of sauroposid β-keratins can be used to differentiate β-keratin matrices from the more widespread and basal α-keratin products [16, 17], as well as from microbial exopolysaccharides [9]. We show here that, contrary to [41, 42], β-keratin tissues may indeed outlast melanin-containing organelles in some cases, and have potential to survive across geological time spans. To be applicable to the fossil record, taphonomic experimental design must take the following factors into account: (1) stabilization of the molecules prior to heating; (2) influence of sediments; (3) potential stabilizing changes occurring when heating occurs more slowly over millions of years; (4) effects of polymerization and increased crosslinks and their ability to protect proteinaceous materials, to accurately approximate processes undergone by keratinous material in naturally occurring conditions. However, we can say with some certainty that any keratin-derived structure that *does* persist into the fossil record has never been exposed to temperatures of 250°C, in combination with pressures of 250 bar, unless it has been first stabilized in early diagenesis by one of these factors [41].

## Supporting information

S1 File (DOCX)

S1 Fig (TIF)

S2 Fig (TIF)

S3 Fig (TIF)

S4 Fig (PDF)

S5 Fig (PDF)

S6 Fig (TIF)
