# Supplementary material for: Preservation potential of keratin in deep time
Source: PLoS One. 2018 Nov 28;13(11):e0206569. doi: 10.1371/journal.pone.0206569 (PMC6261410; doi:10.1371/journal.pone.0206569)
Supplement: S1 File — (DOCX) [file pone.0206569.s001.docx]

Supplemental Materials

**Immunohistochemistry** (**IHC**)

**Materials and methods**

As described in our original study [[1](#_ENREF_1)], rust, grey and white wing feathers and body feathers from a single Hungarian partridge (*Perdix perdix*) were subjected to four environmental conditions spanning a 10-year observation period. Feathers were plucked from a fresh-killed bird, covered to exclude dust, and maintained unaltered at room temperature (*control* feathers). For the other three conditions, feathers from the same bird were buried in fine-grained Judith River (JR) sand collected from a dinosaur excavation site to maintain as much consistency as possible. The burial conditions were; 1) buried in saturated sand in a container with holes for draining, intermittently watered with distilled water (once weekly to monthly on average), and incubated at 60°C for approximately 3 years, then allowed to dry and kept buried at RT for the remaining 7 years of the experiment (*condition 1*); 2) buried in saturated sand, baked at 350°C uninterrupted for 10 years (*condition 2*), then kept at RT, buried, an additional 5 years, when they were collected for analyses in a previous publication ([[1](#_ENREF_1)]). Additional feather fragments from *condition 2* were collected using aseptic protocols and placed in sterile 1.5 ml tubes, sealed and stored at RT until analyzed as described in the current study. For the third condition, ~6–8 inches (~15–20 cm) of JR sands were placed in ~ 1.5m hole dug into native, peat-rich soils in a drainage channel adjacent to a mountain pond. The articulated, freshly killed bird carcass with feathers was placed in the hole and covered with remaining JR sands, then covering with surrounding soil. Although some skeletal elements were recovered, no feathers survived the latter condition, so will not be discussed further.

Upon completion of the experiment (10 years), all feathers were kept covered at room temperature until analysis (~ 7 years for the current study).

Room temperature *control* feathers, *condition 1* (60°C) wet burial feathers, *condition 2* (350°C) dry burial feathers[[1](#_ENREF_1)] and silicified feathers from Yellowstone coot [[2](#_ENREF_2)] were fixed for 1 hour at room temperature in neutral buffered 10% formalin. Fixed feathers were embedded in LR White (hard grade, Electron Microscopy Services) resin blocks after partial dehydration in 70% ethanol.

**Immunofluorescence**

200 nm sections were taken on a Leica EM UC6 Ultramicrotome and transferred to each well of a six-well Teflon coated slide (Electron Microscopy Sciences Cat #63424-06), and dried overnight at 45^o^C. Sections were etched with 25μg/ml Proteinase K in 1X phosphate buffered saline (PBS) buffer at 37^o^C for 15 minutes, followed by 0.5 M ethylenediaminetertraacetic acid (EDTA) pH 8.0 (30 minutes) for antigen retrieval, and lastly with NaBH_4_ (2X10 minutes) for quenching of autofluorescence. Incubations were followed by two five-minute washes in PBS. Sections were then incubated in a blocking buffer of 4% normal goat serum (NGS) in PBS to occupy non-specific binding sites and prevent spurious binding. Primary antibodies (Polyclonal rabbit anti-feather (Biosynthesis BYSN6733, diluted 1:100 in antibody dilution buffer, or dilution buffer alone, to control for non-specific secondary antibody binding. All sections were incubated overnight at 4^o^C. Sections were then incubated with secondary antibody (biotinylated goat anti-rabbit IgG(H+L) (Vector BA-1000) diluted 1:500 for rabbit primary antibody, biotinylated goat anti-chicken IgY (H+L) (Vector BA-9010) diluted 1:500 for chicken primary antibody), for 2 hours at room temperature, followed by incubation with Fluorescein Avidin D (diluted 1:1000, FITC, Vector Laboratories A-2001) for 1 hr at RT. All incubations were separated by sequential washes (2 times for 10 minutes each) in PBS w/Tween 20 followed by two 10-minute rinses in PBS. Finally, sections were mounted with Vectashield H-1000 mounting media, and coverslips were applied. Sections were imaged with a Zeiss Axioskop 2 plus biological microscope and captured using an AxioCam MRc 5 (Zeiss) with 10x ocular magnification on the Axioskop 2 plus in the Axiovision software package (version 4.7.0.0).

**Immunogold Labeling**

We followed a post-embedding TEM immunogold labeling protocol modified from the method shared at IHCWORLD- Life Science Products & Sciences (TEM Immunogold Labeling Protocol-Post-embedding Method Using L.R. White Embedding Medium (<http://www.ihcworld.com/_protocols/em/post_immunoem_l.r.white.htm>). The 90nm sections were collected on carbon -coated nickel grids (EMS Cat CFT200-NI). Grids were incubated on droplets of PBS-Tween 20 for 10 minutes, then normal Donkey serum (NDS) was diluted to 5% in PBS and incubated with the sections for one hour at RT, to occupy non-specific binding sites and prevent spurious binding. Grids were incubated on droplets of primary antibody (Polyclonal Rabbit anti-feather 1:10; polyclonal Chicken anti-avian melanosome 1:20) in primary dilution buffer for 3 hours at room temperature. Sections were rinsed for 10x2 min by placing grids on large droplets of TBS-Tween. All grids were then incubated for one hour with secondary antibodies (12 nm Colloidal Gold AffiniPure Donkey Anti-Rabbit IgG (H+L) 1:20 (Jackson Immuno Research Inc Cat 711-205-152 ) for rabbit primary antibody, or 18 nm Colloidal Gold AffiniPure Donkey Anti-Chicken IgG (H+L) 1:20 (Jackson Immuno Research Inc. Cat 703-215-155) for chicken primary antibody). Grids were rinsed with PBS-Tween20 for 10x2 minutes, followed by 3x30 rinses with distilled water to remove unbound antibodies, then stained with uranyl acetate for 5 minutes and lead citrate for 8 minutes to increase contrast. The sections were observed using the Titan G2 80-200 FEI electron microscope in AIF of North Carolina State University.

**Time of Flight Secondary Ion Mass Spectrometry (ToF-SIMS)**

Prior to ToF-SIMS analysis, the two feather samples (each comprising a ca 5 mm long piece of the rachis) were first pressed between two pieces of aluminium foil, making inner surfaces of the feathers, which are less susceptible to exogenous contamination, accessible for analysis. Whereas the 350 °C feather was easily crushed into particles, the control feather was only flattened by this treatment. The samples were then fixed on silicon substrates using double-sided tape for analysis. A focused, high energy (primary) ion beam was directed onto the sample surface, causing secondary ions to be emitted, resulting in mass spectra containing molecular information. Spatial information was obtained by scanning the primary ion beam over a specified analysis area and recording separate mass spectra in each pixel, allowing the generation of ion images that display the signal intensity of specific molecular signals across the analysis area or of mass spectra of specific regions of interest. It is important to note that the ToF-SIMS spectra are generated from the outermost 1-10 nm of the sample surface and that all molecular species present in the probed analysis area (or region of interest) contribute to the spectra, i.e., there is no chemical separation.

ToF-SIMS analyses were conducted under static SIMS conditions in a TOFSIMSIV instrument (IONTOF GmbH) using 25 keV Bi_3_^+^ primary ions and low-energy electron flooding for charge compensation, with the instrument optimized for high mass resolution (m/Δm ≈ 5,000).

Supplemental references cited:

1. Moyer AE, Zheng W, Schweitzer MH. Keratin durability has implications for the fossil record: results from a 10 Year feather degradation experiment. PLoS One. 2016;11(7):e0157699.

2. Channing A, Schweitzer MH, Horner JR, McEneaney T. A silicified bird from Quaternary hot spring deposits. Proceedings of the Royal Society B-Biological Sciences. 2005;272(1566):905-11. PubMed PMID: ISI:000229929300004.

Supplemental Figure Captions

**S1 Fig.** **In situ immunohistochemistry controls**. Overlay (A, C, E, G) and FITC (B, D, F, H) image of RT *control* (A, B), 60^o^C *condition 1* (C,D), 350^o^C *condition 2* (E, F) and Yellowstone coot feathers ( (E, F), with no primary antibody added, but all other conditions identical to those described for text Figure 3. This assay controls for spurious binding of either the secondary antibody or the fluorescent label, used in these experiments. No binding is visualized to any feather matrix under identical data collection parameters, supporting specificity of the primary anti-chicken feather antisera used in this study.

**S2 Fig.** **Transmission Electron micrographs** at low (A, C) and higher (B,D) magnifications of the condition 2 feathers (A, B) and Yellowstone coot feathers (C, D). Internal microstructures consistent with pith are observed, and patterns of thin walled open pith and thicker ‘junctions’ containing electron-opaque fibrous material (arrows) are visualized. Laminae can be seen in the walls of the Yellowstone feathers (C, D). Magnifications as indicated.

**S3 Fig. Negative ion ToF‐SIMS data of control (RT) feather**. (a) Ion images showing the signal

intensity distributions of the indicated ions. (b) Mass spectra generated from the green and red

regions of interest (ROIs), respectively, as indicated in the lower right ion image in (a). The regularly

spaced peaks at m/z 290‐460 are assigned to monoester molecular ions of the preen wax, with

molecular formulas CnH(2n‐1)O2‐, n=19‐30. The dominating peaks in the low mass range correspond to

CN‐ (m/z 26), CNO‐ (m/z 42), SO3‐ (m/z 80), and HSO4 ‐ (m/z 97). (c) Mass spectrum from the red ROI showing peaks that are tentatively assigned to molecular ions of diesters of the preen wax. The labels indicate the total number of carbon atoms in the two monoester units, linked together with a C3H4 unit in the diester molecules.

**S4 Fig. Positive ion ToF-SIMS data of the 350 °C feather**. (a) Ion images showing the signal intensity distributions of the indicated ions. (b) Mass spectra generated from the green and red regions of interest (ROIs), respectively, as indicated in the lower right ion image in (a). Note the increased signal intensity of the m/z 70 and m/z 30 peaks in the green ROI spectrum compared to the red ROI spectrum. The major peaks in the spectra are assigned to C_2_H_3_^+^ (m/z 27), CH_2_N^+^ (m/z 28), C_2_H_5_^+^ (m/z 29), CH_4_N^+^ (m/z 30), C_3_H_3_^+^ (m/z 39), C_3_H_5_^+^ (m/z 41), C_2_H_4_N^+^ (m/z 42), C_3_H_7_^+^ (m/z 43), C_2_H_6_N^+^ (m/z 44), C_4_H_7_^+^ (m/z 55), C_4_H_8_N^+^ (m/z 70), C_6_H_5_^+^ (m/z 77), and C_7_H_7_^+^ (m/z 91).

**S5 Fig. ToF-SIMS spectra of control (RT), 350 °C feathers, and keratin reference sample**. (a) Negative ions, note the strong signal from N-containing fragment ion peaks in the 350 °C feather spectrum. (b) Positive ions, note the broad distribution of peaks at m/z 100-500 in the 350 °C feather spectrum.

**S6 Fig. Images of the samples analysed by ToF-SIMS**. (a) Light microscopy image of the control feather. Images of the (b) control feather and (c) the 350 C feather (condition 2) taken during ToF-SIMS analysis.
