# Supplementary figures and images for: Preservation potential of keratin in deep time
Source: PLoS One. 2018 Nov 28;13(11):e0206569. doi: 10.1371/journal.pone.0206569 (PMC6261410; doi:10.1371/journal.pone.0206569)

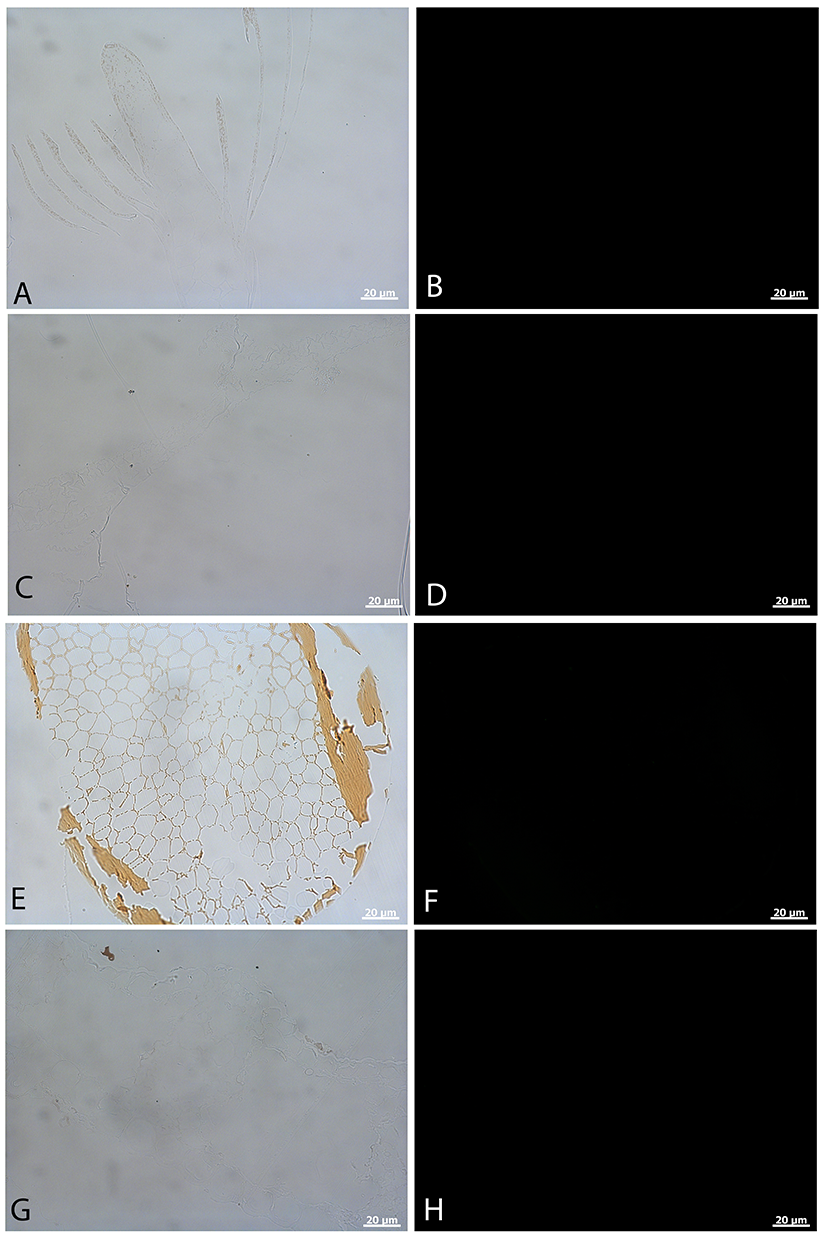

Supplement: S1 Fig — (TIF) [file pone.0206569.s002.tif]

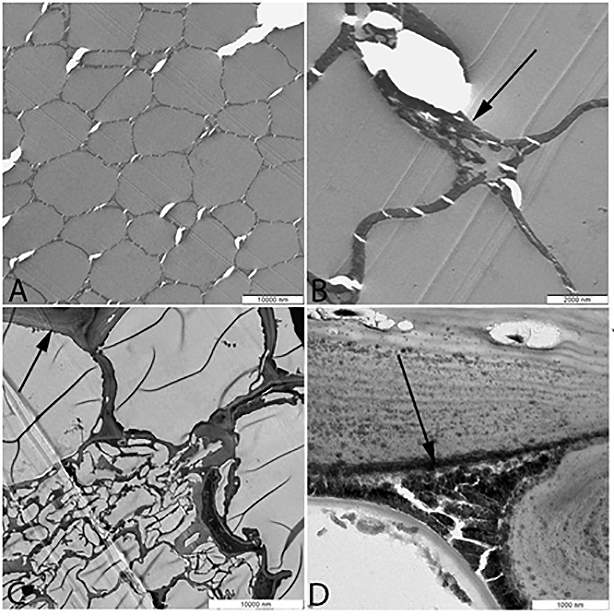

Supplement: S2 Fig — (TIF) [file pone.0206569.s003.tif]

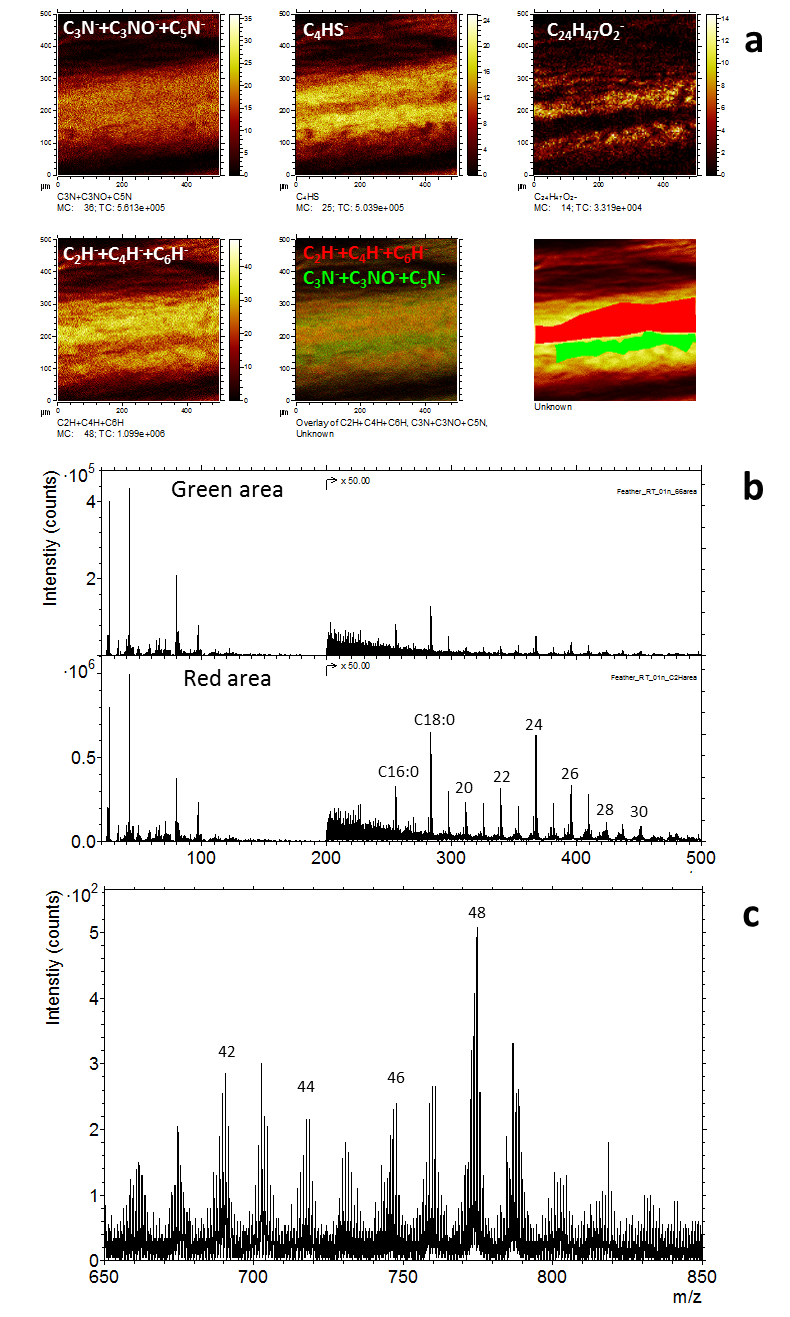

Supplement: S3 Fig — (TIF) [file pone.0206569.s004.tif]

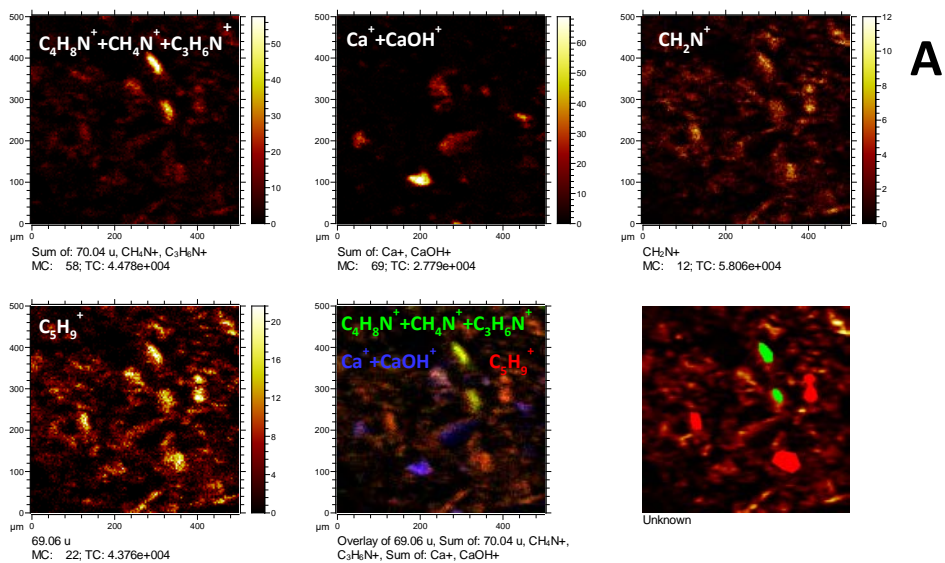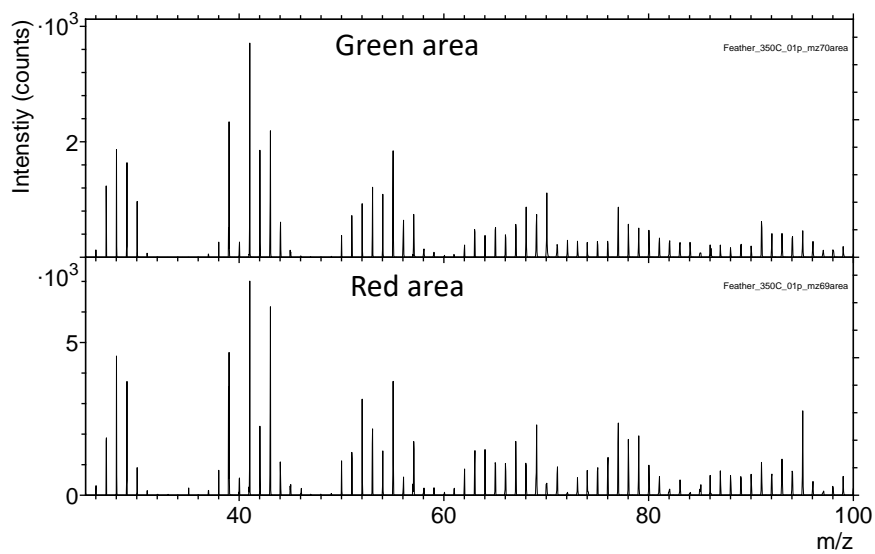

Supplement: S4 Fig — (PDF) [file pone.0206569.s005.pdf]

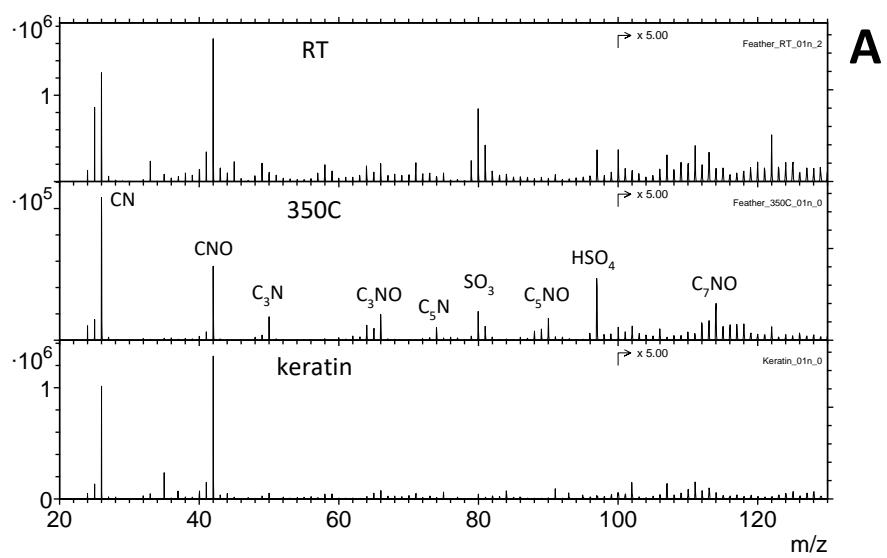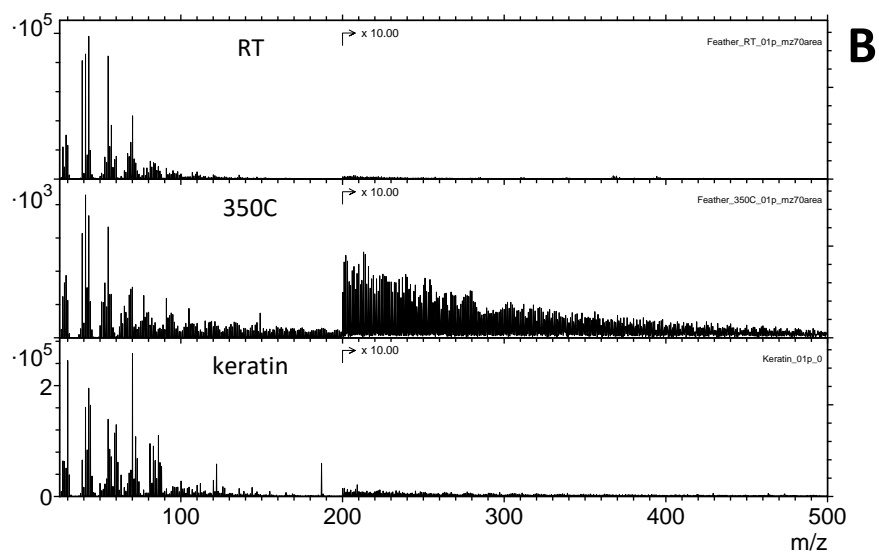

Supplement: S5 Fig — (PDF) [file pone.0206569.s006.pdf]

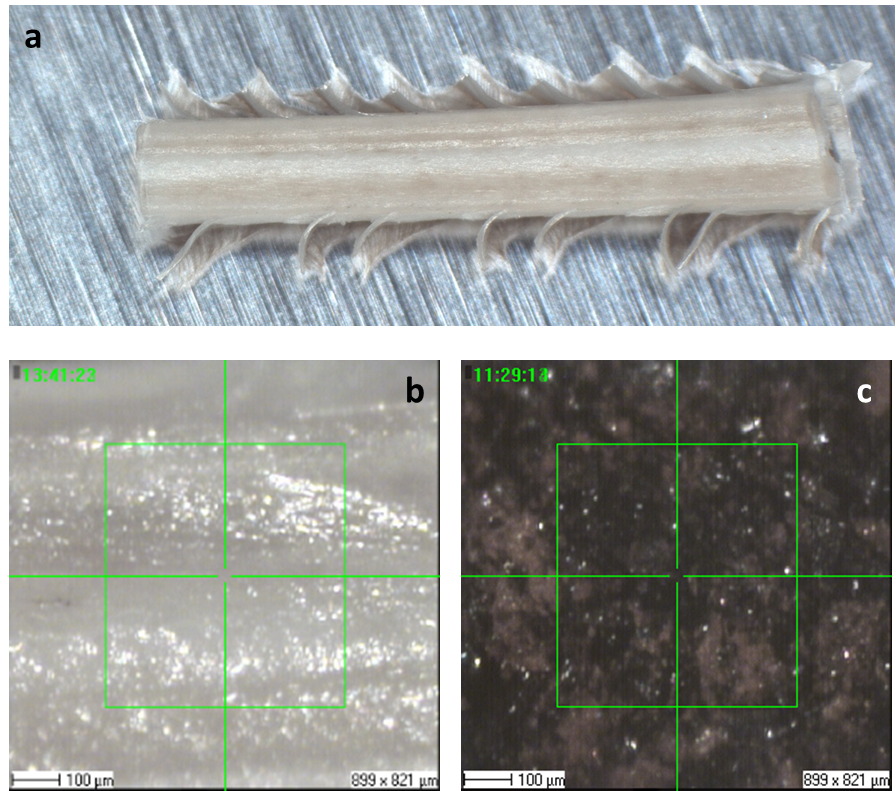

Supplement: S6 Fig — (TIF) [file pone.0206569.s007.tif]
